# Construction of 3D Cellular Composites with Stem Cells Derived from Adipose Tissue and Endothelial Cells by Use of Optical Tweezers in a Natural Polymer Solution

**DOI:** 10.3390/ma12111759

**Published:** 2019-05-30

**Authors:** Takehiro Yamazaki, Toshifumi Kishimoto, Paweł Leszczyński, Koichiro Sadakane, Takahiro Kenmotsu, Hirofumi Watanabe, Tomohiko Kazama, Taro Matsumoto, Kenichi Yoshikawa, Hiroaki Taniguchi

**Affiliations:** 1 Faculty of Life and Medical Sciences, Doshisha University, Kyoto 610-0394, Japan; t.yamazaki13lifephsy@gmail.com (T.Y.); kishimotodoushisha@gmail.com (T.K.); sadakanekoichiro@gmail.com (K.S.); tkenmots@mail.doshisha.ac.jp (T.K.); keyoshik@mail.doshisha.ac.jp (K.Y.); 2 Institute of Genetics and Animal Breeding, Polish Academy of Sciences, Jastrzebiec, 05-552 Magdalenka, Poland; p.leszczynski@ighz.pl; 3 Department of Functional Morphology, Division of Cell Regeneration and Transplantation, Nihon University School of Medicine, Tokyo 173-8610, Japan; watanabe.hirofumi@nihon-u.ac.jp (H.W.); kazama.tomohiko@nihon-u.ac.jp (T.K.); matsumoto.taro@nihon-u.ac.jp (T.M.)

**Keywords:** mesenchymal stem cells, endothelial cells, three-dimensional assemblies, composites with different cell types, depletion effect of polymer

## Abstract

To better understand the regulation and function of cellular interactions, three-dimensional (3D) assemblies of single cells and subsequent functional analysis are gaining popularity in many research fields. While we have developed strategies to build stable cellular structures using optical tweezers in a minimally invasive state, methods for manipulating a wide range of cell types have yet to be established. To mimic organ-like structures, the construction of 3D cellular assemblies with variety of cell types is essential. Our recent studies have shown that the presence of nonspecific soluble polymers in aqueous solution is the key to creating stable 3D cellular assemblies efficiently. The present study further expands on the construction of 3D single cell assemblies using two different cell types. We have successfully generated 3D cellular assemblies, using GFP-labeled adipose tissue-derived stem cells and endothelial cells by using optical tweezers. Our findings will support the development of future applications to further characterize cellular interactions in tissue regeneration.

## 1. Introduction

Mesenchymal stem cells (MSCs) are self-renewing and multi-potent progenitor cells. They differentiate into various mesodermal cell types including adipocytes, osteocytes, and chondrocytes [1]. MSCs are relatively safe, and their clinical use is expanding [2,3,4]. Recent studies have demonstrated that organ-specific interactions between MSCs and endothelial cells play an important role in vascular formation [5]. Interestingly, three-dimensional (3D) cell culture has been performed with MSCs [6]. Kolesky et al. successfully generated pseudo-blood vessels using a mixture of mesenchymal stem cells, vascular endothelial cells, and fibroblasts [7]. Three-dimensional cultures of MSCs lead to chondrogenic differentiation and the production of a matrix for cartilage repair [8]. These findings suggest that the 3D structure of MSCs as well as their interaction with other cells types play important roles in governing their function. Nonetheless, cell-to-cell interactions between MSCs and other cell types within 3D structures have yet to be comprehensively explored.

To generate 3D cellular assemblies in a microenvironment, the development of a methodology that allows for the manipulation of single cells is vital to expanding our understanding of cellular interactions [9,10,11,12,13,14]. Establishing this methodology will provide further insight into the relationship between 3D cellular structures and functions. Thus far, we have successfully established an experimental system to characterize single-cell 3D placement using optical tweezers [15]. Generally, cell–cell contact is inhibited by repulsive interactions, such as electrostatic repulsion, arising from the surface charge of cells [16], short-range repulsion from the hydration structures on the membrane surface [17], and steric repulsion arising from membrane undulation [18,19]. Nonetheless, in diluted polyethylene glycol (PEG) solution, cell pairs stably adhere when they are forced to make contact for more than five minutes using optical tweezers [15]. In the study, stable 3D cellular assemblies were generated by adhering cells one after another. Accordingly, the depletion interaction [20] derived from the entropic effect of PEG molecules likely acts as an attractive force, which overpowers the repulsive forces between cells [15]. Furthermore, natural polymers, including dextran (DEX), stabilize 3D cellular assemblies due to the depletion force [21,22]. In this manner, we have established a nearly non-invasive cell construction system using optical tweezers and the depletion effect of polymers. Nevertheless, methods for manipulating a wide range of cell types have yet to be established. To mimic cellular interactions between a variety of cell types, generating 3D cellular assemblies using different two cell types is essential. Using our methodology, stable 3D cellular assembly can be constructed from different cell types through a mild treatment process. As minimal physical stress is applied to the cells by using natural biopolymer solutions, including DEX, this methodology is superior to other biofabrication techniques such as 3D bioprinting in generating stable 3D cellular assemblies.

In this study, we demonstrate that two different cell types, MSCs and endothelial cells, are located at desired positions in a 3D cellular assembly by using optical tweezers. Accordingly, this methodology is expected to provide a novel approach to characterize the interactions between MSCs and endothelial cells, as well as contribute to the development of regenerative medicine.

## 2. Materials and Methods

### 2.1. Cell Culture

Adipose-derived mesenchymal stem cells (ASCs: Green Fluorescent Protein (GFP)) and Mile Sven 1 (MS1: ATCC) cells were cultured in Dulbecco’s modified Eagle’s medium (DMEM) (Wako Pure Chem. Inc., Osaka, Japan) supplemented with 10% fetal bovine serum (FBS) (Cell Culture Biosci., Nichirei Biosci. Inc., Tokyo, Japan) and 1% penicillin streptomycin (P/S) (Life Tech. Corp., Carlsbad, CA, USA). The cells were cultured at 37 °C in a humidified atmosphere of 5% CO_2_. Subconfluent cells were harvested using trypsin (0.25% Trypsin-EDTA (1X)) (Life Tech. Corp., Carlsbad, CA, USA). Dextran (DEX) (200,000; molecular biology-grade, Wako Pure Chem. Inc., Osaka, Japan) was used to prepare the polymer solution. When appropriate, cells were cultured in DMEM solution containing 40 mg/mL of DEX. The GFP-ASCs were prepared from an adult GFP-mouse (C57BL/6-Tg(CAG-EGFP)10sb/J: Riken BRC) using a previously described method [23]. Ethical approval was provided by the Animal Research and Care Committee at the Nihon University School of Medicine.

### 2.2. Optical Tweezers

**Figure 1:**
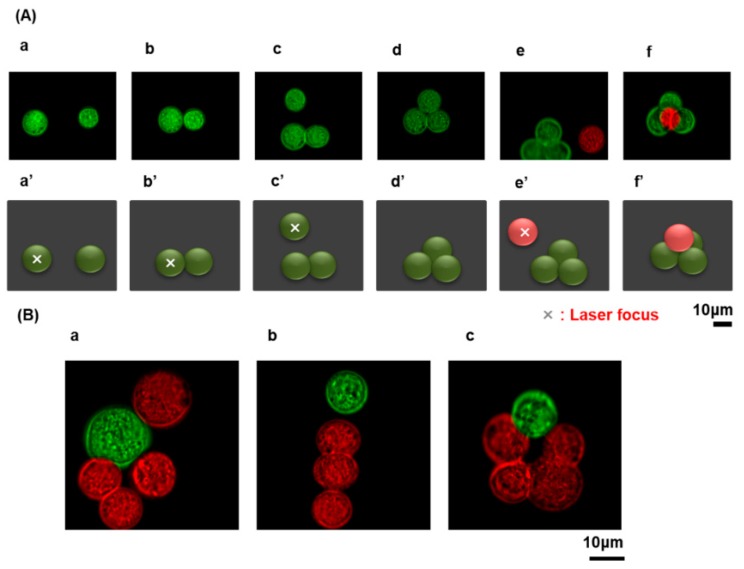
3D assembly of ASCs and MS1 cells. (**A**) Procedure of the optical construction of a pyramidal assembly in a medium with dextran, DEX, (40 mg/mL). (a-f) Microscopic image on the process for the formation of a 3D cell assembly of ASCs (green) and MS1 (red) cells. (a’-f’) The corresponding schematic representation, where X indicates laser focus. (**B**) Various steric arrangements of ASCs and MS1 cells; a, b and c correspond to the shapes of ‘b’, ’i’ and ‘o’, respectively.

**Figure 2:**
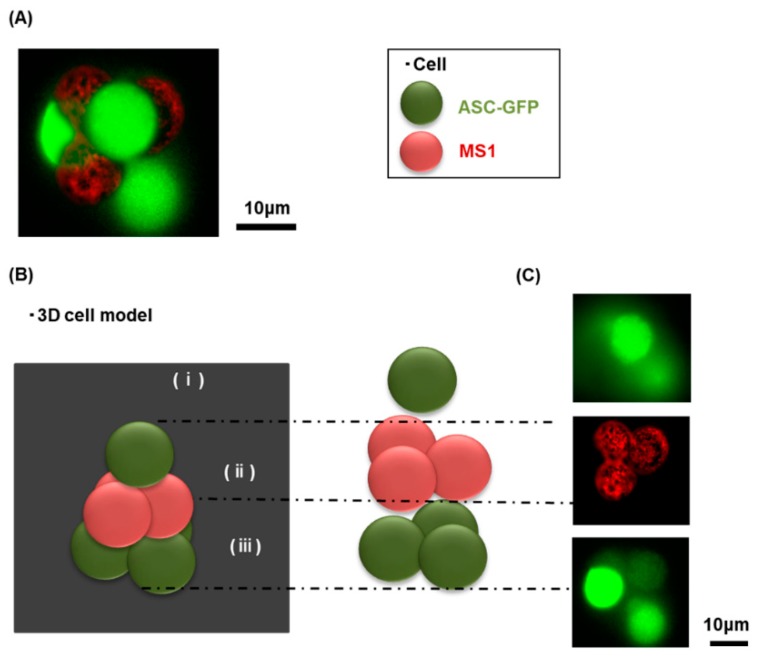
MS1 cell-layer sandwiched by ASCs constructed with laser manipulation in DEX (40 mg/mL) solution. (**A**): Microscopic image on the whole morphology, (**B**): Schematic representation, (**C**): Microscopic image focused on each layer.

**Figure 3:**
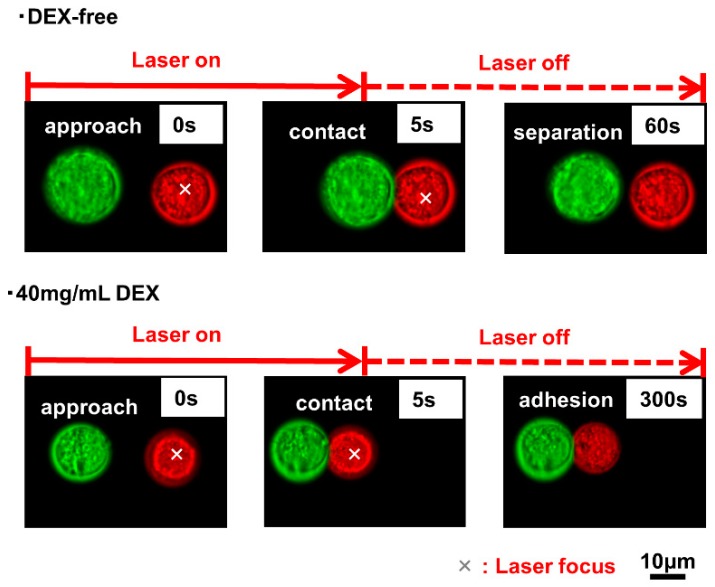
Attachment of a pair of ASCs and MS1 cells by laser manipulation and its stability after switch-off the laser, comparison between in the absence and in the presence of DEX. Upper: Cells in culture medium without DEX, Lower: Cells in culture medium containing 40 mg/mL DEX.

**Figure 4:**
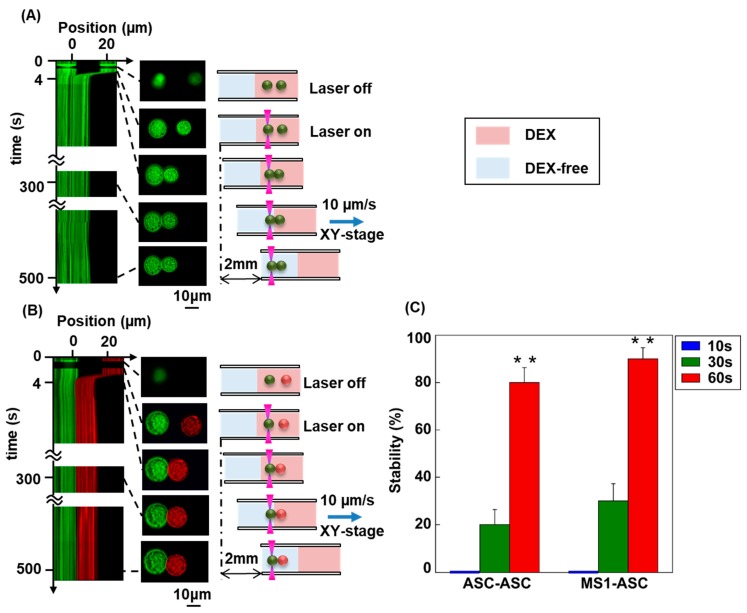
Examination of the stability of cell-cell contact by the transportation onto DEX-free solution after the formation of the contact by Laser manipulation for a pair of ASCs and MS1 cells. (**A**): ASCs-ASCs, (**B**): ASCs-MS1. The cellular pairs were transported onto the region of DEX-free solution at a speed of 10 μm/s after the contact for the period of 300s. Left most: Spatio-temporal diagram, Middle: Fluorescence microscopic image, Right: schematic representation. (**C**): The probability on the formation of stable cell–cell attachments, after the cellular contact for a period of 10, 30 and 60 s, respectively. Error bars represent the standard error of the mean calculated from ten independent measurements.

Optical trapping with a double laser beam was carried out using a commercial optical tweezer instrument (NanoTracker 2, JPK Instruments, Berlin, Germany), which was attached to an inverted microscope (IX71, Olympus, Tokyo, Japan) equipped with a charge-coupled device (CCD) camera (DFK 31AF03, The Imaging Source, Taipei, Taiwan). A 1064-nm continuous wave (CW) laser beam was split into two beams by a polarization beam splitter. Both beams were focused on the sample through a water-immersed objective lens (60×, N.A. = 1.2) for independent trapping. This experimental setup is known as a double-beam configuration [24]. One of these focal points could be moved with a piezo-mirror (focal point 1), while the other was fixed at the same position (focal point 2). In the experiments of Figure 1, Figure 2 and Figure 3 and Figure 4A,B, the cells were manipulated using only the laser beam irradiated to focal point 1. On the other hand, both laser beams irradiated to focal points 1 and 2 were used in the experiment of Figure 4C. The laser’s power at each focal point was set between 40–130 mW. All the experiments were carried out at room temperature (i.e., 25 °C). The identification of ASCs (GFP labeled) and MS1 cells was performed by stimulating these cells with blue light (wavelength of 470 nm) through a GFP filter. To prevent autofluorescence and glare, light-emitting diodes (LEDs) were turned off during cell identification. Pseudo-color processing was performed on the figure as well. ImageJ and Inkscape were used for image processing. The acquired microscopic image was converted to eight bits using ImageJ; then, a pseudo-color image was created with ASC-GFP as green and MS1 as red. Pseudo-color processing was performed by superimposing the layer created by mask processing over the 3D structure, which was generated by manipulating cells with optical tweezers in a polymer solution. The total construction time of the cellular assembly was several minutes (data not shown). In our cell manipulation experiments, cell–cell contact was operated by a remote control with optical tweezers in DEX solution. This allowed us to induce the adherence of cells without using high-voltage laser potential. If the power of the near infrared laser used was 100 mW at the focal point, the cell temperature rise was assumed to be ca. 1 °C [25].

### 2.3. Cell Viability

ASCs (GFP) and MS1 cells were cultured in a six-well dish (Thermo Fisher Scientific, Waltham, MA, USA) and treated with 40 mg/mL of DEX. Cell viability was verified with trypan blue staining after 24 h.

### 2.4. Differentiation Assay

For adipogenic differentiation, ASCs were plated in 30-mm dishes (BD Falcon, Franklin Lakes, NJ, USA) at a density of 5 × 10^4^ cells and grown to confluence. Cells were incubated for 3 weeks in DMEM containing 10% FBS, 1 µM of dexamethasone (Sigma–Aldrich, St. Louis, MO, USA), 0.5 mM of isobutylmethylxanthine (Sigma–Aldrich, St. Louis, MO, USA), and 1× of insulin-transferrin-selenium-X (ITS; Invitrogen). ASCs were fixed for 1 h with 4% paraformaldehyde, incubated in 50% ethanol for 3 min, and then stained with Oil red O (Sigma–Aldrich, St. Louis, MO, USA) for 15 min. For osteogenic differentiation, cells were grown to confluence in 30-mm dishes and incubated for 3 weeks in DMEM containing 10% FBS, 100 nM of dexamethasone, 10 mM of β-glycerophosphate (Sigma–Aldrich, St. Louis, MO, USA), and 50 µM of L-ascorbic acid-2-phosphate (Sigma–Aldrich, St. Louis, MO, USA). The induction medium was replaced every three days. At the indicated time points, ASCs were fixed for 1 h using 4% paraformaldehyde and then rinsed with PBS.

## 3. Results and Discussion

Adipocyte-derived mesenchymal stem cells (ASCs) are one of the most popular cells for cell-based therapy as they are relatively easy to obtain and their implantation does not lead to tumor development [26,27,28,29]. More importantly, they can be differentiated into several cell types including adipocytes, chondrocytes, osteoblasts, hepatocytes, and neurons [30,31,32]; see Figure S1. This differentiation capacity, as well as the cytokines and exosomes that can be derived from these cells, has a considerable impact on the state of adjacent cells [33,34,35]. In this study, we successfully generate 2D and 3D cellular assemblies (3D as a pyramidal shape and 2D as the letter indicating “b-i-o”) using ASCs (Green) and MS1 cells (Red) (Figure 1). Three layers of the three-dimensional cellular assembly are shown in Figure 2. In a cellular environment supplemented with 40 mg/mL of DEX solution, the repulsive forces between cells were suppressed. In turn, the attractive forces with the depletion interaction enabled the generation of an arbitrary 3D structure. Endothelial cells of variable origin respond differently in terms of angiogenic properties. Uwamori et al. reported that human umbilical vein endothelial cells (HUVECs) cultured with MSCs have greater angiogenic capacity than bone marrow endothelial cells (BMECs) cultured with MSCs. In this regard, HUVECs are more capable of inducing angiogenesis [5]. In order to study such a phenomenon in detail, it is necessary to observe interactions at the cellular level in a 3D structure environment. We previously established an efficient method of generating a 3D cellular structure using polymers such as PEG and DEX [15,21,22]. Polymer concentrations below the so-called overlap concentration c* are suitable to generate a stable cellular assembly. At such concentrations, stable cell–cell contact is engendered through the repletion effect of polymers. By applying this methodology to generate 3D cellular structures, we constructed a 3D single cellular assembly of MSCs and MS1 cells. This method allowed us to establish a system characterizing the cellular interactions between MSCs and endothelial cells in an aqueous medium without any solid or gel scaffolds. We have adapted the DEX concentration of 40 mg/mL in this study. Altogether, we successfully generated 3D cellular assemblies, using GFP-labeled adipose tissue-derived stem cells and endothelial cells by using optical tweezers. Together, our findings may lead to the development of future applications for the characterization of cellular interactions and tissue regeneration.

A comparison of single-cell manipulation in either the presence or absence of 40 mg/mL of DEX is presented in Figure 3. Under both conditions, the laser’s output was turned off after maintaining the contact state for a few seconds using the laser trap. After the laser’s output was turned off when the cells were in a DEX-free culture environment, neighboring cells tended to eliminate each other due to repulsive cellular interactions. On the other hand, in the cellular environment containing 40 mg/mL of DEX solution, attractive effects due to polymer depletion overcame these repulsive forces, and contact between cells was maintained.

**Figure 5:**
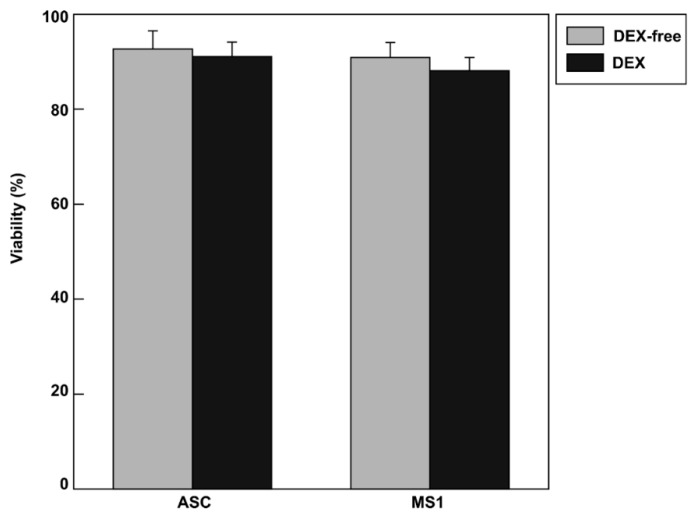
Viability of ASCs and MS1 cells in DEX-free and 40 mg/mL DEX solutions. Error bars represent the standard error of the mean calculated from four independent measurements.

Figure 4A,B present the results of the transport of the cell pair experiment. Cell contact was induced in the polymer solution. Then, the cells were transferred to a culture medium free of macromolecules. The diagram on the left is a spatiotemporal plot. Cell-to-cell contact was maintained for 5 min in the 40 mg/mL of DEX solution after transporting a pair of cells to a culture medium free of DEX and turning off the laser’s output. Figure 4C indicates the stability of the cell tissue body at the time points: 10 s, 30 s, and 60 s. In 40 mg/mL of DEX solution, the cells (ASCs-ASCs/ASCs-MS1 cells) were trapped at focal points 1 and 2, respectively. The contact between these cells was maintained for a few seconds. Thereafter, focal point 2 was fixed in the horizontal direction to verify whether the contact between cells was maintained. When testing two specimens, assuming equal variance, a significant difference was observed in the stability of the cell body when maintaining contact for 60 s at the significance level *P* < 0.01. Similar to previous studies, the viability of ASCs and MS1 cells was not affected by treatment with 40 mg/mL DEX (Figure 5), suggesting that our method successfully generated minimally invasive 3D cellular assemblies. Several studies reported invasive laser manipulation for a variety of cells by using a wide range of lasers [36,37,38]. In the present study, we use a relatively weak laser light of 1064 nm and therefore, cell viability is not impacted by the laser or DEX treatment (Figure 5). The effect exerted by cell-to-cell contact in a macromolecular solution with critical concentration is very different from the osmotic pressure caused by small molecules or ions. The increase in osmotic pressure accompanying the addition of a small chemical species causes cell shrinkage and has an adverse effect on the cell structure. This results in functional deterioration due to water molecule loss through the cell membrane. These results are consistent with previous reports where we built autologous 3D cell assemblies that were generated using 40 mg/mL of PEG [15] and DEX [21,22]. Since cell–cell contacts cannot be achieved in 3D cellular assemblies of two different cell types without a certain concentration of DEX (40 mg/mL, approximate critical concentration), it is assumed that the depletion effect using the critical concentration [21,22] works for heterologous cell types as well. Given the short duration of cellular incubation in our experimental condition, DEX and lasers are unlikely to engender gene expression, including the production of cell adhesion molecules. Accordingly, the timeframe (order of minute) required to generate stable cell–cell contact is shorter than the period of time (order of hour) required to enhance the expression of adhesion molecule. We have previously proposed that an intercellular gap similar to the radius of gyration of high-density polymers occurs between two cells cultured in the presence of a polymer through the depletion interaction [15]. In this regard, the contact area between the neighboring cells increases to cause stable flat cell–cell contact in the presence of polymers such as DEX. Therefore, we believe that DEX plays an essential part in the construction of cellular structures over a short time period on the order of minute. It may be expected that the resulting cell–cell contact through the polymer depletion effect will become tighter under long-term culture conditions due to the expression of adhesion molecules. DEX was used in the culture solution as an example of a polymer with critical concentration. Nonetheless, other water-soluble polymers can be applied in this method in the future. It is also assumed that similar results can be obtained in experimental systems using biological proteins such as albumin or lysozyme. In this case, it is important to adjust the concentration of biological proteins according to their critical concentration. When the polymer exceeds a certain concentration, the macromolecular chains become entangled and the viscosity rapidly increases. When handling cells with optical tweezers in a polymer solution, it is difficult to manipulate cells if the solution is highly viscous. In this study, the overlay concentration of DEX was estimated to be 50 mg/mL based on DEX viscosity measurements [22]; therefore, the concentration of DEX was set at 40 mg/mL. Typically, mesenchymal stem cells are cultured in a special culture medium for several weeks in order to differentiate into adipocytes (Figure S1). In our system, cells are treated with DEX and a laser for several seconds to minutes, and do not differentiate into other cell types, including adipocytes, due to the limited experimental timeframe. Nevertheless, further examination with other natural compounds and longer experimental timeframes should be performed to clarify this point.

## 4. Conclusions

In conclusion, we have successfully generated 3D cellular assemblies, using adipose tissue-derived stem cells and endothelial cells using optical tweezers in the presence of DEX. Using optical tweezers, we have successfully constructed stable 3D cellular assemblies in the presence of DEX and buffer solution. Together with our previous results on the formation of stable cell–cell contact in the presence of solvable macromolecules, our novel methodology is expected to be applicable to various culture cells for the construction of 3D cellular structures. Together, our findings may lead to the development of future applications to further characterize cellular interactions to regenerate tissues.

## Supplementary Materials

The following are available online at https://www.mdpi.com/1996-1944/12/11/1759/s1, Figure S1: Multi lineage potential of mouse ASCs (GFP).

## References

[1] Bianco P., Robey P.G., Simmons P.J. (2008). Mesenchymal Stem Cells: Revisiting History, Concepts, and Assays. Cell Stem Cell.

[2] Alfaifi M., Eom Y.W., Newsome P.N., Baik S.K. (2018). Mesenchymal Stromal Cell Therapy for Liver Diseases. J. Hepatol..

[3] El Agha E., Kramann R., Schneider R.K., Li X., Seeger W., Humphreys B.D., Bellusci S. (2017). Mesenchymal Stem Cells in Fibrotic Disease. Cell Stem Cell.

[4] Lo Furno D., Mannino G., Giuffrida R. (2018). Functional Role of Mesenchymal Stem Cells in the Treatment of Chronic Neurodegenerative Diseases. J. Cell Physiol..

[5] Uwamori H., Ono Y., Yamashita T., Arai K., Sudo R. (2019). Comparison of Organ-Specific Endothelial Cells in Terms of Microvascular Formation and Endothelial Barrier Functions. Microvasc. Res..

[6] Yamamoto K., Tanimura K., Watanabe M., Sano H., Uwamori H., Mabuchi Y., Matsuzaki Y., Chung S., Kamm R.D., Tanishita K. (2018). Construction of Continuous Capillary Networks Stabilized by Pericyte-Like Perivascular Cells. Tissue Eng. Part A.

[7] Kolesky D.B., Homan K.A., Skylar-Scott M.A., Lewis J.A. (2016). Three-Dimensional Bioprinting of Thick Vascularized Tissues. Proc. Natl. Acad. Sci. USA.

[8] Calabrese G., Forte S., Gulino R., Cefali F., Figallo E., Salvatorelli L., Maniscalchi E.T., Angelico G., Parenti R., Gulisano M. (2017). Combination of Collagen-Based Scaffold and Bioactive Factors Induces Adipose-Derived Mesenchymal Stem Cells Chondrogenic Differentiation in Vitro. Front. Physiol..

[9] Cheng Y., Luo X., Tsao C.Y., Wu H.C., Betz J., Payne G.F., Bentley W.E., Rubloff G.W. (2011). Biocompatible Multi-Address 3D Cell Assembly in Microfluidic Devices using Spatially Programmable Gel Formation. Lab. Chip.

[10] Du Y., Lo E., Ali S., Khademhosseini A. (2008). Directed Assembly of Cell-Laden Microgels for Fabrication of 3D Tissue Constructs. Proc. Natl. Acad. Sci. USA.

[11] Fatehullah A., Tan S.H., Barker N. (2016). Organoids as an in Vitro Model of Human Development and Disease. Nat. Cell Biol..

[12] Rossi G., Manfrin A., Lutolf M.P. (2018). Progress and Potential in Organoid Research. Nat. Rev. Genet..

[13] Da Silveira D.S.A., Liberali P. (2018). From Single Cells to Tissue Self-Organization. FEBS J..

[14] Yin X., Mead B.E., Safaee H., Langer R., Karp J.M., Levy O. (2016). Engineering Stem Cell Organoids. Cell Stem Cell.

[15] Hashimoto S., Yoshida A., Ohta T., Taniguchi H., Sadakane K., Yoshikawa K. (2016). Formation of Stable cell–cell Contact without a solid/gel Scaffold: Non-Invasive Manipulation by Laser Under Depletion Interaction with a Polymer. Phys. Lett..

[16] Donath E., Voigt A. (1983). Charge Distribution within Cell Surface Coats of Single and Interacting Surfaces--a Minimum Free Electrostatic Energy Approach. Conclusions for Electrophoretic Mobility Measurements. J. Theor. Biol..

[17] LeNeveu D.M., Rand R.P., Parsegian V.A. (1976). Measurement of Forces between Lecithin Bilayers. Nature.

[18] Sackmann E., Smith A.S. (2014). Physics of Cell Adhesion: Some Lessons from Cell-Mimetic Systems. Soft Matter.

[19] Chen L., Jia N., Gao L., Fang W., Golubovic L. (2013). Effects of Antimicrobial Peptide Revealed by Simulations: Translocation, Pore Formation, Membrane Corrugation and Euler Buckling. Int. J. Mol. Sci..

[20] Asakura S., Oosawa F. (1954). On Interaction between Two Bodies Immersed in a Solution of Macromolecules. J. Chem. Phys..

[21] Yamazaki T., Taniguchi H., Tsuji S., Sato S., Kenmotsu T., Yoshikawa K., Sadakane K. (2018). Manipulating Living Cells to Construct STable 3D Cellular Assembly without Artificial Scaffold. J. Vis. Exp..

[22] Yoshida A., Tsuji S., Taniguchi H., Kenmotsu T., Sadakane K., Yoshikawa K. (2017). Manipulating Living Cells to Construct a 3D Single-Cell Assembly without an Artificial Scaffold. Polymers.

[23] Matsumoto T., Kano K., Kondo D., Fukuda N., Iribe Y., Tanaka N., Matsubara Y., Sakuma T., Satomi A., Otaki M. (2008). Mature Adipocyte-Derived Dedifferentiated Fat Cells Exhibit Multilineage Potential. J. Cell Physiol..

[24] Rauch P., Jähnke T. (2014). Optical Tweezers for Quantitative Force Measurements and Live Cell Experiments. Microsc. Today.

[25] Liu Y., Cheng D.K., Sonek G.J., Berns M.W., Chapman C.F., Tromberg B.J. (1995). Evidence for Localized Cell Heating Induced by Infrared Optical Tweezers. Biophys. J..

[26] Choi S., Ryoo S.B., Park K.J., Kim D.S., Song K.H., Kim K.H., Chung S.S., Shin E.J., Cho Y.B., Oh S.T. (2017). Autologous Adipose Tissue-Derived Stem Cells for the Treatment of Complex Perianal Fistulas Not Associated with Crohn’s Disease: A Phase II Clinical Trial for Safety and Efficacy. Tech. Coloproctol..

[27] Lee J.H., Park C.H., Chun K.H., Hong S.S. (2015). Effect of Adipose-Derived Stem Cell-Conditioned Medium on the Proliferation and Migration of B16 Melanoma Cells. Oncol. Lett..

[28] Scioli M.G., Artuso S., D’Angelo C., Porru M., D’Amico F., Bielli A., Gentile P., Cervelli V., Leonetti C., Orlandi A. (2018). Adipose-Derived Stem Cell-Mediated Paclitaxel Delivery Inhibits Breast Cancer Growth. PLoS ONE.

[29] Veriter S., Andre W., Aouassar N., Poirel H.A., Lafosse A., Docquier P.L., Dufrane D. (2015). Human Adipose-Derived Mesenchymal Stem Cells in Cell Therapy: Safety and Feasibility in Different “Hospital Exemption” Clinical Applications. PLoS ONE.

[30] Baer P.C., Geiger H. (2012). Adipose-Derived Mesenchymal stromal/stem Cells: Tissue Localization, Characterization, and Heterogeneity. Stem Cells Int..

[31] Cawthorn W.P., Scheller E.L., MacDougald O.A. (2012). Adipose Tissue Stem Cells Meet Preadipocyte Commitment: Going Back to the Future. J. Lipid Res..

[32] Ghaedi M., Tuleuova N., Zern M.A., Wu J., Revzin A. (2011). Bottom-Up Signaling from HGF-Containing Surfaces Promotes Hepatic Differentiation of Mesenchymal Stem Cells. Biochem. Biophys. Res. Commun..

[33] Hu L., Wang J., Zhou X., Xiong Z., Zhao J., Yu R., Huang F., Zhang H., Chen L. (2016). Exosomes Derived from Human Adipose Mensenchymal Stem Cells Accelerates Cutaneous Wound Healing Via Optimizing the Characteristics of Fibroblasts. Sci. Rep..

[34] Lee M., Liu T., Im W., Kim M. (2016). Exosomes from Adipose-Derived Stem Cells Ameliorate Phenotype of Huntington’s Disease in Vitro Model. Eur. J. Neurosci..

[35] Nahar S., Nakashima Y., Miyagi-Shiohira C., Kinjo T., Toyoda Z., Kobayashi N., Saitoh I., Watanabe M., Noguchi H., Fujita J. (2018). Cytokines in Adipose-Derived Mesenchymal Stem Cells Promote the Healing of Liver Disease. World J. Stem Cells.

[36] Ayano S., Wakamoto Y., Yamashita S., Yasuda K. (2006). Quantitative Measurement of Damage Caused by 1064-Nm Wavelength Optical Trapping of Escherichia Coli Cells using on-Chip Single Cell Cultivation System. Biochem. Biophys. Res. Commun..

[37] Grover S.C., Skirtach A.G., Gauthier R.C., Grover C.P. (2001). Automated Single-Cell Sorting System Based on Optical Trapping. J. Biomed. Opt..

[38] Keloth A., Anderson O., Risbridger D., Paterson L. (2018). Single Cell Isolation using Optical Tweezers. Micromachines.

