# Supplementary material for: Construction of 3D Cellular Composites with Stem Cells Derived from Adipose Tissue and Endothelial Cells by Use of Optical Tweezers in a Natural Polymer Solution
Source: Materials (Basel). 2019 May 30;12(11):1759. doi: 10.3390/ma12111759 (PMC6601048; doi:10.3390/ma12111759)
Supplement: Supplementary file 1 [file materials-12-01759-s001.pdf]

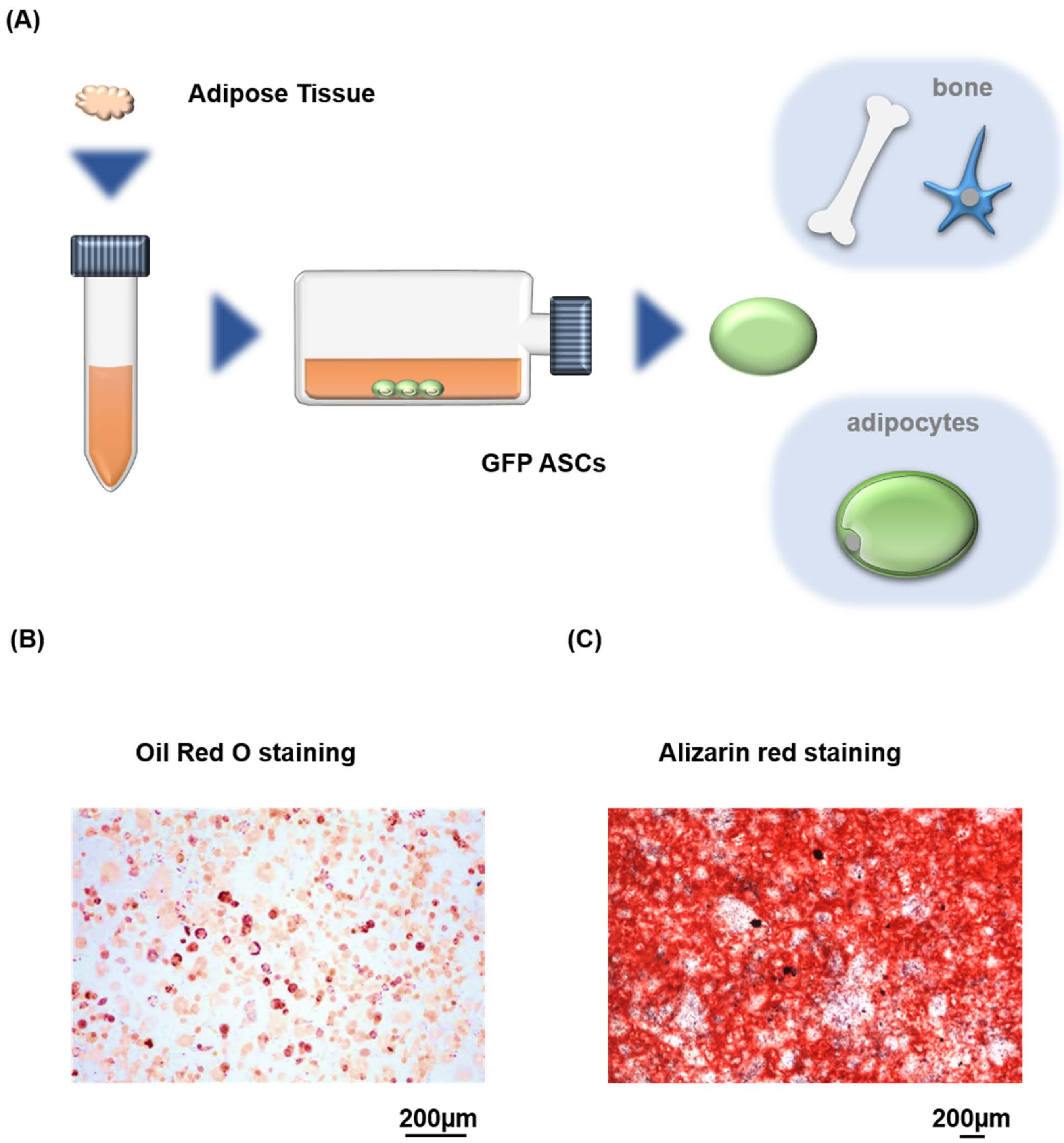


**Figure S1. Multi lineage potential of mouse ASCs (GFP)** Mouse ASCs were cultured for 3 weeks in the appropriate induction media to evaluate their ability to differentiate (A). The cells were analyzed for (B) adipogenic (Oil red-O) and (C) osteogenic (alizarin red S) differentiation capacity.
